# Adipocyte Hyperplasia Facilitated Adipose Tissue Expansion to Alleviate Hepatopancreas Injury in Nile Tilapia (*Oreochromis niloticus*) Fed High-Fat Diet

**DOI:** 10.1155/anu/1260555

**Published:** 2025-07-02

**Authors:** Senyue Tan, Jiamin Wei, Ailan Diao, Douglas R. Tocher, Zeling Lin, Bing Chen, Ruixin Li, Shuqi Wang, Cuiying Chen

**Affiliations:** ^1^Marine Biology Institute of Shantou University and Guangdong Provincial Key Laboratory of Marine Biotechnology, Shantou, Guangdong 515063, China; ^2^Institute of Animal Science, Guangdong Academy of Agricultural Sciences, Collaborative Innovation Center of Aquatic Sciences, Key Laboratory of Animal Nutrition and Feed Science in South China, Guangzhou 510640, China

**Keywords:** glucose metabolism, hepatopancreas, high-fat diet, lipid metabolism, mesenteric adipose tissue, Nile tilapia

## Abstract

Previous studies showed that interplay between liver and adipose tissue was important for animals to adapt to high-fat diets (HFDs). While the mechanisms of adaptation to HFD are not fully understood in fish, we hypothesize that interaction between these key tissues will be crucial. The present study evaluated the physiological and biochemical characteristics and gene expression profiles of hepatopancreas and adipose tissue of Nile tilapia (*Oreochromus niloticus*; initial weight, 20.01 ± 0.01 g) fed diets containing either 6% lipid (normal-fat diet [NFD]) or 12% lipid (HFD) for up to 10 weeks. While growth was not affected, serum and hepatopancreatic lipid contents increased significantly in tilapia fed HFD compared to fish fed NFD at 6 weeks (*p*  < 0.05). In addition, feeding HFD for 6 weeks induced hepatopancreatic injury as shown by increased alanine aminotransferase (ALT) and aspartate aminotransferase (AST) activities in serum and higher expression of genes related to inflammation (*tnfβ* and *il-1β*) and malondialdehyde (MDA) content in hepatopancreas (*p*  < 0.05). However, after feeding HFD for 10 weeks, serum and hepatopancreatic lipid contents and injury indices decreased, whereas mesenteric fat index (MFI) and expression of genes related to glucose (GLU) metabolism (*pfk*, *g6pd*, and *glut2*) in hepatopancreas increased significantly compared to the NFD group (*p*  < 0.05). Significant expansion of mesenteric adipose tissue was observed in tilapia fed HFD, due mainly to adipocyte hypertrophy at 6 and 8 weeks and hyperplasia at 10 weeks. With the expansion of mesenteric adipose tissue, the expression of genes related to lipid metabolism and inflammation increased at 8 weeks, but decreased at 10 weeks. The data indicated that excess dietary lipid accumulated initially in hepatopancreas of tilapia consuming HFD, but prolonged intake promoted mesenteric adipose tissue development, potentially mitigating hepatopancreas damage caused by excess lipid deposition. Additionally, enhanced hepatopancreatic glycolysis may contribute to the adaptation of tilapia to HFD intake.

## 1. Introduction

In modern aquaculture, high-fat diets (HFDs) are used commonly to improve feed efficiency and reduce cost in fish farming by providing high energy and preserving dietary protein for growth [1, 2]. However, HFD feeding in fish often leads to metabolic disorders related to deposition of excess fat in tissues, especially liver and intestine, stressing organelles such as endoplasmic reticulum, mitochondria, and peroxisomes, inducing chronic inflammation, and altering intestinal flora [3–5]. These metabolic disorders can adversely affect growth, immunity, and survival, ultimately reducing the economic returns of fish farming [6–8]. However, there are metabolic adaptive mechanisms that can partly mitigate the physiological injury caused by HFD feeding in fish. For example, Li et al. [9] reported increased cholesterol–bile acid flux that, in turn, stimulated peroxisomal fatty acid β-oxidation, which may be a metabolic adaptation in response to short-term feeding of HFD in tilapia. However, while increased cholesterol–bile acid flow in hepatopancreas may represent a compensatory mechanism, it was ineffective when HFD was fed over an extended period [9]. Therefore, it is imperative to gain a deeper understanding of potential metabolic adaptations in fish fed HFD in order to increase the effectiveness of high-energy diets in aquaculture.

It is well appreciated that the liver is a pleiotropic endocrine organ that is generally the first to receive and process nutrients from the intestine and, subsequently, to influence metabolism in both local and distal organs [10]. High lipid deposition in the liver has been considered to be a strong predictor of insulin resistance and normally precedes the development of other metabolic abnormalities including macrophage infiltration and inflammation of adipose tissue, adipocyte hypertrophy, and cell death [10–14]. As another major endocrine organ, adipose tissue also regulates energy and metabolic homeostasis through the storage and release of free fatty acids and via the secretion of a variety of adipokines [15, 16]. Studies indicated that the balance between hypertrophy (increased size) and hyperplasia (increased number) of adipocytes could affect the metabolic outcome of obesity [17, 18]. If adipose tissue expands primarily through hypertrophy, when adipocytes reach their maximum lipid storage capacity, excess fatty acids may be deposited ectopically in other tissues including liver, further exacerbating metabolic disturbances in the body [19, 20]. Conversely, an increase in the number of adipocytes through adipogenesis can promote healthy expansion of adipose tissue and overall metabolic health [21, 22]. The accumulation of lipid droplets in hepatocytes, which may be the result of several dysfunctions and multiple organ interplays, can eventually lead to hepatic steatosis and liver disease [23]. Thus, the cross talk between liver and other organs, especially adipose tissue, is critical for effective metabolic adaptations to dietary lipid overload and to maintain lipid homeostasis [10, 16].

Depending upon species, liver, adipose tissue, and muscle have the capability to store lipids in fish and, as in mammals, white adipose tissue (WAT) is an important lipid storage site in many teleosts including Nile tilapia (*Oreochromis niloticus*), grass carp (*Ctenopharyngodon idellus*), pomfret (*Brama brama*), large yellow croaker (*Larimichthys crocea*), and turbot (*Scophthalmus maximus*) [24–30]. It was shown that the majority of lipid storage occurs normally within adipose tissue, rather than in hepatopancreas, which would represent an effective strategy for tilapia to adapt to being fed HFD [25]. However, when fish are fed HFD for a prolonged period and lipid intake continues to be high, the storage capacity of adipocytes may reach a limit and, in this context, enhancing the capacity of fish adipose tissue to store lipid could represent an effective solution [25, 31]. Accordingly, Wei et al. [31] employed a Pparγ agonist to induce a hyperplasia-dominant pattern of expansion in the abdominal adipose tissue of grass carp juveniles fed HFD. This altered the normal hypertrophy-dominant pattern of expansion of adipose tissue to a hyperplasia-dominant pattern of expansion, and this “healthier” pattern of lipid storage in adipose tissue was an important contributor to the adaptation of grass carp to HFD [27]. Thus, it appears that the interplay between liver and adipose tissue is important for fish to adapt to HFD and, therefore, maintaining metabolic homeostasis by promoting healthy expansion of adipose tissue could be a potential nutritional strategy to alleviate the negative effects of high-energy diets. However, the metabolic characteristics of liver and other organs, especially the adipose tissue, during HFD feeding are not fully understood in fish. In particular, the metabolic processes and interaction between liver and adipose tissue under different modes of expansion and their underlying cross talk mechanisms in fish fed HFD require further investigation.

Glucose (GLU), as a fundamental energy substrate in mammals that exhibits a protein-sparing effect analogous to lipids, could theoretically serve as an energy source in aquatic feeds. However, its metabolic significance appears limited in teleost species. Extensive studies have documented sustained hyperglycemia in fish fed high-carbohydrate diets, with excessive GLU intake even triggering anorexia, particularly in carnivorous species [32, 33]. While Enes et al. [34] reported that hepatic regulation of GLU storage occurs in most fish species, the differential modulation of glycolytic and gluconeogenic pathways in piscine liver results in inefficient dietary GLU utilization. This metabolic contradiction forms a competition between exogenous GLU and persistently elevated endogenous GLU pools, which may underlie the low dietary carbohydrate utilization efficiency observed in fish [34]. Notably, endogenous GLU remains physiologically indispensable despite this apparent intolerance to exogenous carbohydrates. Polakof et al. [35] demonstrated that carbohydrate intolerance is less pronounced in herbivorous and omnivorous species and, even in carnivorous fish, GLU serves critical functions in specific tissues or metabolic processes.

Nile tilapia (*O. niloticus*), an important aquaculture species worldwide, is an omnivorous model species and, as such, provides a valuable system for investigating adaptive mechanisms of GLU homeostasis during prolonged HFD feeding. In Nile tilapia, two abdominal organs are primarily involved in lipid storage including the hepatopancreas (an integrated organ of liver and pancreas) and mesenteric WAT. In the present study, changes in the physiological and biochemical characteristics of hepatopancreas and mesenteric adipose tissue and the interaction of the two organs in metabolic processes, including underlying cross talk mechanisms, were investigated and compared in juvenile Nile tilapia fed either a normal-fat diet (NFD) or a HFD for up to 10 weeks. The results showed that tilapia exhibited some adaptations to HFD intake, including alteration of lipid distribution pattern with the development of mesenteric adipose tissue and modification in hepatopancreatic lipid and GLU metabolism. The findings suggested potential strategies to enhance the adaptability of farmed fish to prolonged HFD intake and improve feed utilization efficiency.

## 2. Materials and Methods

### 2.1. Animal Ethics Statement

All experimental procedures were performed in compliance with the guidelines for the care and use of animals as recommended by the Institutional Animal Care and Use Committee of Shantou University (No. 132, revised July 16, 2022; Shantou, China).

### 2.2. Experimental Animals, Feeds, and Feeding Trial

Nile tilapia juveniles were obtained from the San'ao Freshwater Fish Hatchery of Jieyang City (Guangdong Province, China) and acclimated for 2 weeks in an indoor recirculating water system at Shantou University, during which time they were fed a commercial feed (32% protein and 6% lipid). At the initiation of the feeding experiment, 180 juveniles with an average initial mass of 20.01 ± 0.01 g were allocated randomly into six 160 L tanks with 30 fish per tank. Three tanks were fed a diet with standard “normal” fat level (6% lipid, NFD) and three tanks were fed a diet with a high fat level (12% lipid, HFD) and, thus, the tank (cage) was the experimental unit. The formulations and proximate compositions of the two experimental diets are presented in Table 1. During the 10-week feeding trial, all tanks were supplied with feed twice daily at 8:00 and 16:00 with the daily feeding ration decreasing from 5% to 3% of body weight as the fish grew. Feces and uneaten feed residues were removed in a timely manner to maintain water quality. The experiment was performed in a recycled aquaculture system (RAS) with a flow rate of 15 L per min using freshwater (0 ppt) with ambient temperature between 25.0 and 28.0°C, pH between 7.0 and 7.4, dissolved oxygen level more than 7.0 ppm, and ammonia-nitrogen concentration below 0.01 ppm.

### 2.3. Growth Performance and Sampling

Fish in each tank were bulk weighed every 2 weeks to alter feed ration (see above) and, in addition, the tilapia were sampled after 6, 8, and 10 weeks of feeding. At these samplings, three fish from each tank were collected randomly and anesthetized with 0.1‰ tricaine methane sulfonate (MS222) solution and their lengths and weights measured. From the recorded parameters, feed conversion ratio (FCR), feed intake (FI), final body weight (FBW), initial body weight (IBW), survival rate, specific growth rate (SGR), and weight gain rate (WGR) were calculated as described previously [8]. Blood was collected immediately from the tail vein using a 1 mL syringe, allowed to stand at room temperature for 1 h, and then, centrifuged at 3500 × *g* for 10 min at 4°C. The collected supernatant (serum) was stored at −80°C for later biochemical analysis. The viscera, hepatopancreas, and abdominal (mesenteric) fat were then excised immediately from the same fish and weighed for the calculation of viscerosomatic index (VSI), hepatosomatic index (HSI), and mesenteric fat index (MFI) as described in detail previously [9]. Samples of hepatopancreas, mesenteric adipose tissue, and dorsal muscle were also collected for further analysis. For biochemical composition and gene expression, tissue samples were snap frozen in liquid nitrogen and stored at −80°C prior to further processing and analyses. Samples of hepatopancreas and abdominal adipose tissue were also collected and stored in 4% paraformaldehyde at room temperature prior to tissue sectioning and histological analysis and determination of tissue lipid deposition and content.

### 2.4. Proximate Compositions of Feeds

The moisture contents of feeds were determined by drying in an oven at 105°C until constant weight. The dried diets were pulverized using a pestle and mortar and protein determined by the Kjeldahl method (Foss Kjeltec 8400 automatic analyzer, Foss, Denmark, Europe). Ash contents of feeds were determined by incineration in a muffle furnace (CWF111513216P1, Carbolite Gero, Derbyshire, UK) at 550°C for 5 h. Lipid contents of feeds and tilapia dorsal muscle were determined by the ether-extraction method using a soxhlet extractor (SOX606, Haineneg, Beijing, China) [36].

### 2.5. Physiological and Biochemical Indicators in Serum and Hepatopancreas

The levels of GLU, triglyceride (TG), total cholesterol (TC), and nonesterified fatty acid (NEFA) in serum and hepatopancreas were determined by the methods of hexokinase, glycerol-3-phosphate oxidase-peroxidase (GPO-PAP), cholesterol oxidase-peroxidase amino-antipyrine (CHOD-PAP), and acyl-CoA synthetase-acyl coenzyme A oxidase-peroxidase (ACS-ACOD-POD), respectively. The activity of aspartate aminotransferase (AST) and alanine aminotransferase (ALT) in serum were detected by the methods of lactate dehydrogenase-ultraviolet (LDH-UV) and malate dehydrogenase-ultraviolet (MDH-UV), respectively. The content of malondialdehyde (MDA) and total protein in hepatopancreas were determined by the methods of thiobarbituric acid (TBA) method and allophanamide, respectively. The kits were acquired from Nanjing Jiancheng Bioengineering Institute and the assay of the serum and hepatopancreas indicators were performed using a fully functional microplate detector (H1MF, Biotek Synergy, America, The USA).

### 2.6. Histological Analysis and Oil Red O (ORO) Staining

Tilapia hepatopancreas and mesenteric adipose tissues (*n* = 3 per tank) were stored and fixed in 4% paraformaldehyde solution for 48 h, then, dehydrated through graded ethanol concentrations, paraffin-embedded and sectioned (5 μm) for hematoxylin and eosin (H&E) staining. In addition, ORO staining of tilapia hepatopancreatic tissues was performed in the following steps: 4% paraformaldehyde fixation for 48 h, tissue freezing, optimal cutting temperature (OCT) compound embedding, sectioning (5 μm), and staining. Stained sections were observed under a light microscope (Axio Imager 2, Zess, Germany) and images were captured by a camera (Axiocam 506, Zeiss) and acquired by software (ZEN 2, Zeiss). Lipid droplets stained with ORO and adipocyte surface area stained with HE were quantified using Image-pro plus version 6.0 (Media Cybernetics, Maryland, MD, USA).

### 2.7. Tissue RNA Isolation and First Strand Complementary DNA (cDNA) Synthesis

Total RNA was extracted from hepatopancreas, mesenteric adipose tissue, and dorsal muscle using VeZol Reagent according to the manufacturer's instructions (Vazyme Biotech, China). The quality and concentration of extracted RNA were determined using NanoDrop One Spectrophotometer (Thermo Fisher Scientific Inc., USA) with an OD260/OD280 ratio of total RNA between 1.8 and 2.2 and a weight more than 5 μg. The RNA integrity was confirmed by 1.0% agarose gel electrophoresis. Then first-strand cDNA was synthesized using HiScript Ⅳ RT SuperMix for quantitative PCR (qPCR; +gDNA wiper) reagent following the manufacturer's protocol (Vazyme Biotech).

### 2.8. Quantitative Real-Time PCR

Transcript levels of genes related to inflammation, lipid, and GLU metabolism were determined in hepatopancreas, dorsal muscle, and adipose tissue by SYBR Green-based real-time qPCR using primers as shown in Table S1. All assays were carried out on the LightCycler 480 thermocycler (Roche, Sweden) in accordance with the manufacturers' instructions, with 10 μL total reaction volumes containing 5 μL of ChamQ Universal SYBR qPCR Master Mix (Vazyme Biotech), 2 μL of diluted cDNA, 0.2 μL each of forward and reverse primers (10 mM), and 2.6 μL of ultrapure water. The amplification procedure was as follows: 95°C for 5 min, followed by 40 cycles of denaturation at 95°C for 10 s, annealing, and extension at 60°C for 30 s, with 1 cycle at 95°C for 15 s, 60°C for 60 s, and 95°C for 15 s to produce melting curves to check the specificity of the primers. Each sample was run in triplicate and reactions without templates were used as negative controls. The relative transcript levels of each gene were normalized with that of *18s rRNA* gene and calculated via the 2^−*ΔΔ*Ct^ method.

### 2.9. Statistical Analysis

Results were presented as means ± SEM (standard error of the mean) with *n* = 3 or as indicated and each experiment was repeated three times. The normality of data distribution was assessed using the Kolmogorov–Smirnov test and the homogeneity of variance was evaluated using Bartlett's test. Based on these tests, parametric statistical methods were deemed appropriate for the analysis. To evaluate the effects of diets (NFD and HFD) and feeding duration (6, 8, and 10 weeks) on grow and physiological/biochemical parameters, a two-way ANOVA was performed. In cases where significant interactions were detected, post hoc analyses were conducted using Duncan's test for multiple group comparisons. For comparisons between the two diet groups, independent sample Student's *t*-tests were applied. To control for the increased risk of Type I errors due to multiple comparisons, a Bonferroni correction was applied where appropriate. All statistical analyses and graphical representations were performed using GraphPad Prism 8.0 (GraphPad Software, San Diego, California, USA). A *p* value < 0.05 was considered statistically significant and a *p* value < 0.01 was considered highly statistically significant. Asterisks (two diets), different lowercase superscript letters (treatment groups), and uppercase superscript letters (feeding duration) were applied to mean values in the Tables and Figures to indicate significant differences between the two diets, or among the treatment groups (i.e., diet × duration combinations), or the duration of feeding, respectively.

## 3. Results

### 3.1. Growth Performance and Serum Biochemical Indices of Tilapia Fed NFD and HFD Diets

At the end of the trial, all fish appeared healthy and survival rate was not related to diet. Irrespective of diet, FBW and WGR increased and FI and SGR decreased, as feeding time increased (*p* < 0.05), while no significant differences were detected between tilapia fed HFD and NFD in growth performance including FBW, WGR, FI, SGR, and FCR (Table 2). However, FBW, WGR, FCR and SGR, and the organ indices VSI, HSI, and MFI showed significant interactions between diet and feeding time (*p* < 0.05, Tables 2 and 3). As feeding time increased, VSI and HSI gradually decreased, while MFI increased significantly, with highest HSI and MFI observed in fish fed HFD at 6 and 10 weeks, respectively (*p* < 0.05, Table 3). Furthermore, HSI and MFI were higher in tilapia fed HFD compared to fish fed NFD at 6 and 10 weeks, respectively (*p* < 0.01, Table 3). There were no significant differences in CF of fish among the treatments (Table 3).

Serum biochemical indices of tilapia fed HFD and NFD for 6, 8, and 10 weeks are shown in Table 4. According to two-way ANOVA, serum TG, TC, NEFA, and GLU levels, and AST and ALT activities showed significant interactions between dietary lipid content and feeding time (*p* < 0.05, Table 4). Serum TG and TC showed increasing trends, while NEFA showed a significant decreasing trend, from 6 to 10 weeks in tilapia fed NFD (*p* < 0.05, Table 4). In contrast, in fish fed HFD, serum TG, TC, and NEFA levels all decreased with feeding duration and were significantly lower at 10 weeks compared to 6 weeks (*p* < 0.05, Table 4). Serum GLU showed a significant increasing trend in fish fed both diets from 6 to 10 weeks, with highest level observed in fish fed NFD at 10 weeks (*p* < 0.05, Table 4). Serum AST and ALT activities were elevated considerably in tilapia fed HFD compared to fish fed NFD, significantly so at 6 weeks with AST, and at all-time points with ALT (*p* < 0.01, Table 4). In addition, serum AST activity showed a decreasing trend in fish fed both diets and was significantly lower at 8 and 10 weeks compared to 6 weeks in fish fed HFD (*p* < 0.05, Table 4). These data indicated hepatopancreas damage in Nile tilapia fed HFD for 6 weeks and that the degree of damage decreased with prolonged feeding of HFD.

### 3.2. The Response to HFD was Earlier in Hepatopancreas Than in Adipose Tissue

**Figure 1:**
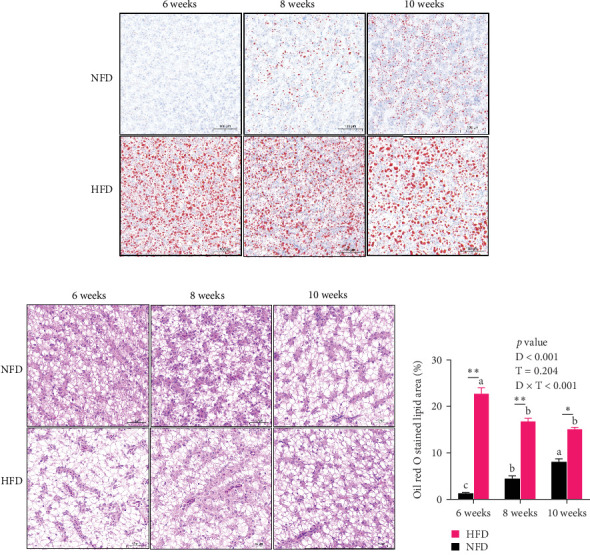
Lipid deposition in hepatopancreas of Nile tilapia fed NFD and HFD for 6, 8, and 10 weeks. (A) Oil red O, (B) hematoxylin–eosin staining of hepatopancreas, and (C) the quantification of lipids droplet content of hepatopancreas strained by oil red O (area/total cell area), scale bars: 100 μm. Data are presented as means ± SEM (*n* = 3). D, diets; D × F, interaction between diets and feeding time; F, feeding time; HFD, high-fat diet; NFD, normal-fat diet. Asterisks indicate a significant difference between fish fed HFD and fish fed NFD, *⁣*^*∗*^*p* < 0.05, *⁣*^*∗∗*^*p* < 0.01. Different letters on columns for the same diet indicate significant differences due to duration of feeding (*p* < 0.05).

Oil red staining showed clearly that the content of lipid droplets was considerably greater in tilapia fed HFD compared to fish fed NFD, but that lipid droplet numbers increased from 6 to 10 weeks in fish fed NFD, while they declined in fish fed HFD (Figure 1A). H&E-stained hepatopancreas showed a similar pattern with marginal vacuolation much greater at 6 weeks in tilapia fed HFD compared to fish fed NFD and that vacuolation increased with increased feeding time in fish fed NFD, while it decreased in fish fed HFD (Figure 1B). Quantification of oil red staining showed that the area of lipid deposition was significantly greater in tilapia fed HFD compared to fish fed NFD at all-time points, but that the area increased significantly in fish fed NFD and decreased significantly in fish fed HFD from 6 to 10 weeks (*p* < 0.05, Figure 1C). Irrespective of feeding duration, TG, TC, NEFA, and GLU levels of hepatopancreas were generally significantly higher in fish fed HFD compared with fish fed NFD (*p* < 0.05, Table 5). Hepatopancreatic TG, TC, NEFA, and GLU levels from 6 to 10 weeks decreased initially and then, increased in fish fed HFD (*p* < 0.05), but none of the differences were statistically significant in fish fed NFD (Table 5). The levels of MDA generally followed the same pattern as TG, TC, and NEFA, being higher at 6 weeks in fish fed HFD than in fish fed NFD, but then decreased significantly in fish fed HFD so that it was at a similar level to those in fish fed NFD at 8 and 10 weeks (*p* < 0.05, Table 5).

**Figure 2:**
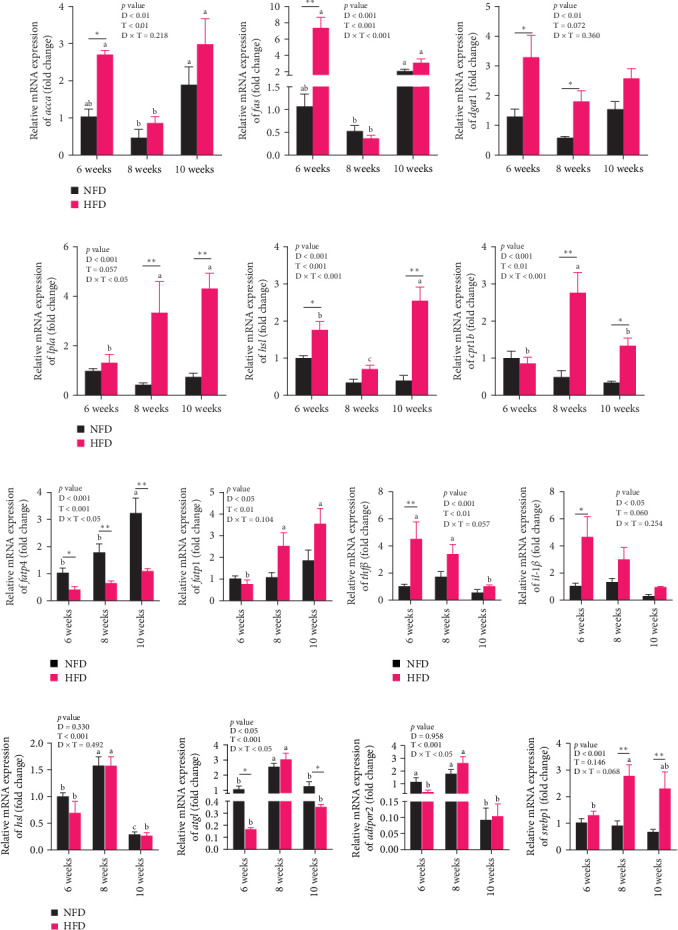
The relative expression levels of genes related to lipid metabolism and inflammation in hepatopancreas and adipose tissue of Nile tilapia fed NFD and HFD for 6-, 8-, and 10-week. (A) Hepatopancreatic lipid biosynthesis-related genes (*accα*, *fas*, and *dgat1*), (B) hepatopancreatic lipolysis-related genes (*lplα*, *hsl*, and *cpt1b*), (C) hepatopancreatic lipid transport-related genes (*fatp4* and *fabp1*), (D) hepatopancreatic inflammatory cytokine genes (*tnfβ* and *il-1β*), and (E) lipid metabolism-related genes (*hsl*, *atgl*, *adipor2*, and *srebp1*) in adipose tissue. D, diets; D × F, interaction between diets and feeding time; F, feeding time; HFD, high-fat diet; NFD, normal-fat diet. Data are presented as means ± SEM (*n* = 3). Asterisks indicate a significant difference between fish fed HFD and fish fed NFD, *⁣*^*∗*^*p* < 0.05, *⁣*^*∗∗*^*p* < 0.01. Different letters on columns for the same diet indicate significant differences due to duration of feeding (*p* < 0.05). *accα*, acetyl-CoA carboxylase alpha; *adipor2*, adiponectin receptor 2; *atgl*, adipose triglyceride lipase; *cpt1b*, carnitine palmitoyl transferase 1b; *dgat1*, diacylglycerol O-acyltransferase 1; *fabp1*, fatty acid binding protein 1; *fas*, fatty acid synthase; *fatp4*, fatty acid transport protein 4; *hsl*, hormone-sensitive triglyceride lipase; *il-1β*, interleukin-1 beta; *lplα*, lipoprotein lipase alpha; *srebp1*: sterol regulatory element-binding protein 1; *tnfβ*, tumor necrosis factor beta.

The expression of genes related to lipid metabolism and inflammatory cytokines in hepatopancreas were significantly affected by dietary lipid content (*p* < 0.05, Figure 2A–C). The lipogenesis genes, *accα*, *fas*, and *dgat1*, were significantly higher at 6 weeks in hepatopancreas of fish fed HFD compared to fish fed NFD (Figure 2A, *p* < 0.05). Irrespective of diet, the expression of these genes tended to decrease at 8 weeks, before increasing again at 10 weeks. Expression levels of the lipolysis-related genes, *lplα*, *hsl*, and *cpt1b*, in hepatopancreas showed significant interaction between dietary lipid level and feeding time (*p* < 0.05) and were generally significantly higher in fish fed HFD compared to fish fed NFD (*p* < 0.05), especially at 10 weeks, with expression levels tending to show an upward trend in fish fed HFD and a downward trend in fish fed NFD from 6 to 10 weeks (Figure 2B). The fatty acid transport genes *fatp4* and *fabp1* showed significant differences with feeding duration and increased significantly at 8 and 10 weeks in fish fed both HFD and NFD (*p* < 0.05, Figure 2C). Expression levels of *fatp4* showed significant interaction between dietary lipid content and feeding time and were significantly lower, while levels of *fatbp1* were numerically (but not statistically) higher in fish fed HFD compared to fish fed NFD. Hepatopancreas expression levels of the inflammatory factors, *tnfβ* and *il-1β*, were elevated significantly at 6 weeks in tilapia fed HFD compared to fish fed NFD, but decreased significantly from 6 to 10 weeks in fish fed HFD, while levels remained relatively unchanged over this period in fish fed NFD (Figure 2D).

In mesenteric adipose tissue, expression levels of lipid metabolism-related genes, *hsl*, *atgl*, and *adipor2*, at 6 weeks tended to be lower, significantly so in the case of *atgl*, in fish fed HFD compared to fish fed NFD (Figure 2E). Irrespective of diet, the expression of these genes and *srebp1* in adipose tissue increased at 8 weeks, before decreasing at 10 weeks, with most differences being significant. Expression of *srebp1* at 8 and 10 weeks was significantly higher in fish fed HFD compared to fish fed NFD (*p* < 0.05, Figure 2E). To put the above changes in lipid metabolism in hepatopancreas and adipose tissue into further context, lipid content and metabolism were also investigated in skeletal muscle. Lipid content of dorsal muscle was constant throughout the experiment and was not affected significantly by either diet or duration of feeding (Figure S1). Furthermore, while the expression levels of several lipid metabolism-related genes showed increasing trends with duration of feeding and were higher at 10 weeks than at 6 weeks, significantly so for several genes, diet had no significant effect other than higher expression of *lpl* at 10 weeks in tilapia fed HFD compared to fish fed NFD (Figure S2).

Overall, the results indicated that lipid accumulated first in the hepatopancreas followed by the mesenteric adipose tissue in tilapia fed HFD (Tables 2 and 3 and Figure 1) and that lipid deposition and changes in expression of lipid metabolism-related genes in adipose tissue lagged behind those in hepatopancreas (Figure 2A,C,E), indicating the initial metabolic response to feeding HFD was in hepatopancreas.

### 3.3. Increased Gluconeogenesis and Glycolysis Relieved the Excess Lipid Deposition in Hepatopancreas Caused by Feeding Tilapia HFD

**Figure 3:**
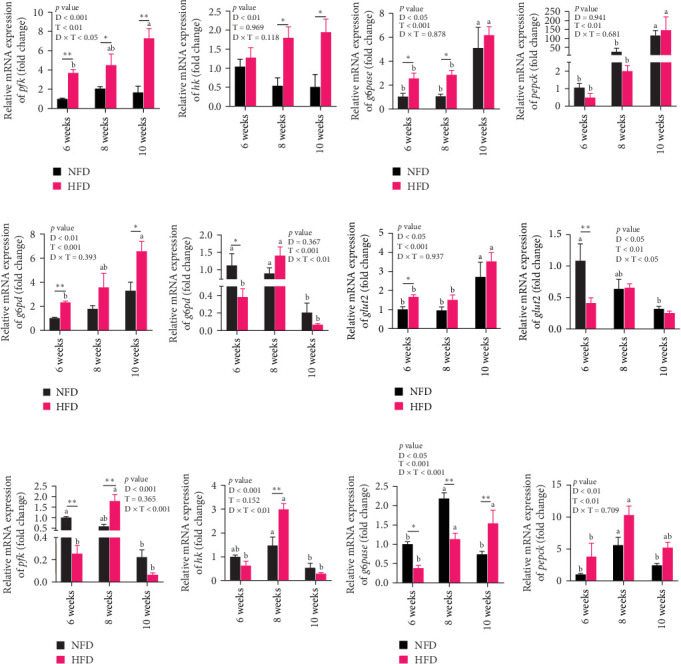
The relative expression of glucose metabolism-related genes in hepatopancreas and mesenteric adipose tissue of Nile tilapia fed NFD and HFD for 6-, 8-, and 10-week. (A, E) Genes related to glycolysis (*pfk* and *hk*) in hepatopancreas and mesenteric adipose tissue, respectively. (B, F) Genes related to gluconeogenesis (*g6pdase* and *pepck*) in hepatopancreas and mesenteric adipose tissue, respectively. (C) Gene related to pentose phosphate pathway (*g6pd*) in hepatopancreas and mesenteric adipose tissue, respectively. (D) Gene related to glucose transport (*glut2*) in hepatopancreas and mesenteric adipose tissue, respectively. D, diets; D × F, interaction between diets and feeding time; F, feeding time; HFD, high-fat diet; NFD, normal-fat diet. Data are presented as means ± SEM (*n* = 3); asterisks indicate significant differences between fish fed HFD and NFD at the same duration of feeding, *⁣*^*∗*^*p* < 0.05, *⁣*^*∗∗*^*p* < 0.01. Different letters on columns for the same diet indicate significant differences due to duration of feeding (*p* < 0.05); *g6pase*, glucose-6-phosphatase; *g6pd*, glucose-6-phosphate dehydrogenase; *glut2*, glucose transport 2; *hk*, hexokinase; *pepck*, phosphoenolpyruvate carboxy kinase; *pfk*, phosphofructokinase.

As GLU levels in serum and hepatopancreas increased with feeding duration, especially in tilapia fed HFD, the relative abundance of GLU metabolism-related genes was investigated in hepatopancreas and adipose tissue. The expression of key genes associated with glycolysis (*pfk*), gluconeogenesis (*g6pase* and *pepck*), the pentose phosphate pathway (*g6pd*), and GLU transport (*glut2*) in hepatopancreas were affected significantly by both dietary lipid level and feeding time (*p* < 0.01, Figure 3). Expression levels of these genes increased with duration of feeding in tilapia fed HFD, with levels of all the genes elevated significantly at 10 weeks compared to 6 weeks (*p*  < 0.05, Figure 3A–D). The hepatopancreas expression levels of gluconeogenesis-related genes, *g6pase*, *pepck*, and *g6pd*, and GLU transporter *glut2*, showed similar increasing trends with increased feeding duration in fish fed NFD (Figure 3B–D). Moreover, the expression levels of *pfk*, *g6pase*, *g6pd*, and *glut2* in hepatopancreas were significantly higher in fish fed HFD compared to fish fed NFD (Figure 3A–D). The increased hepatopancreatic expression of glycolytic and gluconeogenic genes and the increased level of serum GLU with prolonged consumption of HFD suggested that increased gluconeogenesis and glycolysis may play some role in the alleviation of excessive lipid deposition in hepatopancreas and, if so, be a mechanism of adaptation to HFD intake in tilapia.

As shown in Figure 3C–F, the expression levels of *g6pd*, *glut2*, *pfk*, *hk*, and *g6pase* in mesenteric adipose tissue showed significant interaction between dietary lipid level and feeding time (*p* < 0.05, Figure 3C–F). In contrast to hepatopancreas, the expression levels of *g6pd*, *glut2*, and *g6pase* at 6 weeks were significantly lower in tilapia fed HFD compared to fish fed NFD (*p*  < 0.05, Figure 3C–F). However, expression levels of *pfk*, *hk*, *g6pase*, *pepck*, and *g6pd* in adipose tissue increased significantly in fish fed HFD at 8 weeks, after which expression levels decreased at 10-weeks (*p*  < 0.05, Figure 3C,E,F). A similar pattern of expression, increased at 8 weeks and then decreased at 10 weeks, was observed in *g6pase* and *pepck* in fish NFD (*p*  < 0.05, Figure 3F). However, expression levels of *g6pd*, *glut2*, and *pfk* showed a consistent decreasing trend in fish fed NFD, with significantly lower expression at 10 weeks compared to 6 weeks (Figure 3C–E). The expression of genes related to GLU metabolism showed the generally opposite pattern in adipose tissue compared to hepatopancreas, suggesting interplay between these tissues in GLU metabolism that may play an important mechanistic role in maintaining serum GLU levels during adaptation to prolonged HFD intake in Nile tilapia.

### 3.4. HFD Induced Mesenteric Adipose Tissue Development, Which in Turn Alleviated Hepatopancreas Injury Induced by Prolonged HFD Feeding in Nile Tilapia

**Figure 4:**
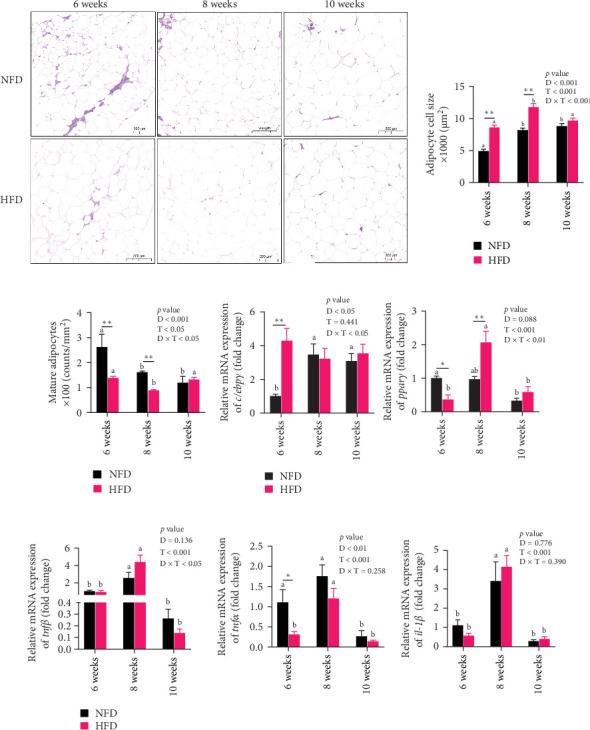
Morphology and relative expression of genes associated with differentiation, inflammation, and lipid metabolism in mesenteric adipose tissue of Nile tilapia fed NFD and HFD for 6-, 8-, and 10-week. (A) Hematoxylin–eosin staining of adipose tissue. (B, C) Adipocyte cell size and number, respectively. (D) Genes related to differentiation (*c/ebpγ* and *pparγ*). (E) Genes related to inflammation (*tnfβ*, *tnfα*, and *il-1β*). D, diets; D × F, interaction between diets and feeding time; F, feeding time; HFD, high-fat diet; NFD, normal-fat diet. Data are presented as means ± SEM (*n* = 3); asterisks indicate significant differences between fish fed HFD and NFD at the same duration of feeding, *⁣*^*∗*^*p* < 0.05, *⁣*^*∗∗*^*p* < 0.01. Different letters on columns for the same diet indicate significant differences due to duration of feeding (*p* < 0.05); *c/ebpγ*, CCAAT/enhancer binding protein gamma; *il-1β*, interleukin-1 beta; *pparγ*, peroxisome proliferator-activated receptor gamma; *tnfα*, tumor necrosis factor alpha; *tnfβ*, tumor necrosis factor beta.

To gain further insight into the development of adipose tissue in tilapia under different dietary conditions, the morphological and proliferative-metabolic characteristics of mesenteric adipose tissue were investigated. Staining by HE and quantitative measurement of the size and number of adipocytes in mesenteric adipose tissue showed that both dietary lipid level and feeding duration significantly affected adipocyte size and number and there was significant interaction (Figure 4A–C). Adipocytes were significantly larger at 6 and 8 weeks in fish fed HFD than in fish fed NFD, while, conversely, adipocyte number was significantly greater in fish fed NFD than fish fed HFD (*p* < 0.01, Figure 4A–C). By 10 weeks, there were no differences in adipocyte size and number between fish fed HFD and NFD, due to size decreasing and cell number increasing with duration of feeding in fish fed HFD and the opposite happening in fish fed NFD. Thus, the increased mesenteric adipose tissue mass (MFI as proxy) in fish fed HFD compared to fish fed NFD (Table 2) was caused mainly by hypertrophy at Weeks 6 and 8, with hyperplasia only contributing at Week 10 (Figure 4A–C). In contrast, MFI was lower in fish fed NFD and did not increase significantly until Week 10 (Table 2), with the increase due entirely to hypertrophy of adipocytes (Figure 4A–C).

Two-way ANOVA analysis showed that expression of *c/ebpγ*, involved in adipocyte differentiation, was significantly higher in adipose tissue of fish tilapia fed HFD compared to fish fed NFD (*p* < 0.05, Figure 4A). Furthermore, expression of *c/ebpγ* in adipose tissue was significantly higher at 6 weeks in fish fed HFD compared to fish fed NFD, while the level of *c/ebpγ* of fish fed NFD increased significantly at 8 and 10 weeks to be similar to that of fish fed HFD (Figure 4D). Together, these data suggested that HFD promoted the differentiation and development of adipose tissue in tilapia juveniles. Consistent with this, while the expression of *pparγ*, a key transcription factor in lipid metabolism and adipocyte differentiation was significantly higher at 6 weeks in fish fed NFD compared to fish fed HFD (*p* < 0.05), it was higher in fish fed HFD at the latter two time points, significantly so at 8 weeks (*p*  < 0.01, Figure 4D). The pattern of expression of *pparγ* in fish fed HFD, being significantly higher at 8 weeks than at either 6 or 10 weeks, was similar to the pattern observed in lipid metabolism-related genes (Figure 3E) and was also repeated in the expression patterns of the inflammatory factors *tnfα*, *tnfβ*, and *il-1β* that also showed significantly highest expression at 8 weeks compared to 6 and 10 weeks in fish fed HFD (*p* < 0.05) with fish fed NFD showing a qualitatively similar pattern (Figure 4E). These results indicated that the expansion of mesenteric adipose tissue induced by HFD was mainly by adipocyte hypertrophy and accompanied by inflammation in Weeks 6–8, while inflammation was reduced significantly by 10 week when hyperplasia may play a relatively greater role. This may be the reason why lipid deposition did not continue to increase, but gradually decreased in hepatopancreas of tilapia with increasing duration of HFD intake.

## 4. Discussion

The liver is a key organ in energy metabolism and immunity and serves as the principal mediator of biological responses to a multitude of adverse external stimuli [2, 37]. Previous studies have shown that, in addition to issues such as viral infection and water quality, feed quality, quantity, and nutrient composition are major factors affecting liver health in fish [5, 38–41]. Among the latter, high-energy diets have been demonstrated to induce liver damage, primarily through increased deposition of lipid, lipid peroxidation, and inflammation [1, 3, 41]. The present study revealed that hepatopancreatic lipid deposition and inflammation were increased in tilapia fed HFD at 6 weeks, while, as the fish grew, the lipid deposition and inflammation in hepatopancreas did not increase further. This was different from previous studies [24] and we suggest that this may be associated with the quantity of feeding. As in commercial culture, feeding levels of around 3%–5% of body weight were chosen to provide the nutrients required for tilapia growth without overfeeding. This experimental design was conducive to inducing the adaptive mechanism of tilapia to HFD without directly causing serious damage beyond the body's regulatory capacity. Accordingly, lipid accumulation was greater in hepatopancreas than in adipose tissue of Nile tilapia after short-term (6 weeks) feeding of HFD. This suggested that the hepatopancreas was the first organ in tilapia to respond to lipid overload, followed by adipose tissue, which was consistent with the situation in mice [10]. Moreover, expression levels in hepatopancreas of genes related to lipid biosynthesis were higher and genes related to lipolysis were lower, at 6-week in fish fed HFD compared with fish fed NFD, indicating a lipogenic status that promoted/supported lipid accumulation in hepatopancreas.

The lipid overload in hepatopancreas at 6 weeks was accompanied by increased cell vacuolization and inflammation in fish fed HFD compared to fish fed NFD, suggesting that mitigating these physiological and biochemical changes in hepatopancreas during HFD feeding could be an approach to alleviating the health risks of high-energy diets in tilapia. In this respect, reduction of hepatic NEFA flow in HFD feeding was demonstrated to be an effective strategy for ameliorating ectopic lipid deposition in liver [42]. The NEFA pool in liver can be derived from three sources: dietary intake, de novo lipogenesis and lipolysis, and NEFA can be either catabolized for energy or esterified into lipid for storage or to supply peripheral tissues with nutrients [43]. The latter suggested that increased esterification of hepatopancreatic NEFA into lipids in fish fed HFD could be ultimately beneficial to hepatopancreatic health. In the present study, hepatopancreatic and serum NEFA levels of tilapia decreased after 8 and 10 weeks of HFD feeding, which may be one reason why hepatopancreas inflammation and vacuolation reduced with prolonged HFD intake. At the same time, it was interesting that GLU levels in hepatopancreas and serum showed an increasing trend as feeding duration increased, perhaps hinting that reduced hepatopancreas NEFA and increased GLU metabolism may be related indirectly. Irrespective of precise mechanism, it indicated that Nile tilapia exhibit a range of adaptive responses to HFD intake and that regulation of GLU metabolism, in addition to lipid metabolism, may play a role in this adaptation.

The metabolic balance of GLU and lipid that can both serve as major sources of energy is a crucial prerequisite for maintaining the physiological activity of metabolic organs [44]. It was demonstrated previously that enhanced glycogen utilization in cardiomyocytes represented an effective strategy for mitigating the progression of cardiac hypertrophy in mice fed HFD [45]. However, GLU and lipid metabolism are two different pathways and lipid catabolism does not produce gluconeogenic precursors in animals, other than a small amount of glycerol that can be converted to GLU via gluconeogenesis to maintain blood GLU homeostasis during starvation [46–48]. However, it is clear that feeding HFD affects GLU metabolism, mainly via regulating activities of insulin-related pathways [9, 49]. Therefore, Liu et al. [49] reported that the role of leptin was selective in diet-induced obesity models in Nile tilapia, with leptin activating GLU metabolism, but showing leptin resistance in lipid metabolism regulation in fish fed HFD, while it promoted lipid metabolism and showed leptin resistance in GLU metabolism regulation in fish fed a high-carbohydrate diet (HCD). In the present study, there was a notable upregulation in the expression of genes related to GLU uptake, gluconeogenesis/glycogen synthesis, and glycolysis in the hepatopancreas of tilapia fed HFD. Thus, increased gluconeogenesis and serum GLU level was balanced by a corresponding increase in glycolysis. Increased hepatopancreatic GLU metabolism may be a mechanism to enable/facilitate the storage and utilization of lipid in other organs, especially adipose tissue, the primary lipid storage organ in tilapia, which, in an evolutionary context, enabled the fish to store (and utilize) more energy. Irrespective of diet, lipid was deposited initially in hepatopancreas, but that expansion of mesenteric adipose tissue started sooner and was more rapid in tilapia fed HFD than fish fed NFD. Whatever, enhanced glycogen utilization in hepatopancreas could contribute to the alleviation of hepatopancreatic lipid overload in fish fed HFD and this may be one of the mechanisms by which Nile tilapia adapt to long-term HFD feeding. However, the underlying mechanisms require further investigation.

Previous research demonstrated that the expansion of mesenteric adipose tissue induced by Pparγ agonist was an effective strategy for attenuating liver injury induced by HFD feeding in grass carp [31]. He et al. [25] also reported that feeding HFD (12% lipid) to Nile tilapia for 10 weeks significantly increased adipose tissue mass, with no significant difference in HSI and lipid deposition in hepatopancreas compared with fish fed a low-fat diet. Similarly, in the present study, mesenteric adipose tissue exhibited a notable increase in volume after 8–10 weeks of HFD feeding. The expansion of adipose tissue at 10 weeks in tilapia fed HFD occurred concurrently with increased hepatopancreatic lipolysis and the alleviation of lipid overload and degree of injury (AST and ALT) of hepatopancreas. This suggested that the expansion of mesenteric adipose tissue in response to HFD may be a mechanism mitigating hepatopancreatic injury under conditions of lipid overload. However, adipose tissue has the capacity to expand in two distinct modes: hypertrophy and hyperplasia [27, 31], and it has been postulated that a predominantly proliferative model of expansion is healthier [50]. Several studies have demonstrated that establishing a healthy expansion pattern of adipose tissue, especially visceral adipose tissue, can be an effective way to alleviate the progression of nonalcoholic fatty liver disease (NAFLD) in mice fed HFD [41, 51–54]. In the present study, mesenteric adipose tissue exhibited disparate expansion patterns in response to the two dietary regimens. The development of mesenteric adipose tissue was predominantly hypertrophy at 6 weeks in tilapia fed HFD, but, as feeding continued, hypertrophy decreased and hyperplasia became relatively more important by 10 weeks. Additionally, lipid and GLU metabolic activity and adipose tissue inflammation were elevated at 8 weeks, followed by the suppression of lipolytic metabolism and a reduction in serum NEFA levels at 10 weeks. The opposite pattern was observed in fish fed NFD, with expansion of mesenteric adipose tissue caused predominantly by hyperplasia at 6 weeks, then principally by hypertrophy at 10 weeks. However, while a previous study reported that adipose tissue exhibited hyperplasia and became the primary site for lipid accumulation in Nile tilapia fed HFD for 10 weeks [25], there is a general lack of research and data on the dynamics of adipose tissue expansion patterns in tilapia.

Studies in mammals have shown that in obesity, WAT undergoes comprehensive cellular and structural remodeling to better adapt to nutrient input and enhance energy storage capacity [50]. Initially, tissue expansion occurs through adipocyte hypertrophy or hyperplasia [55]. Subsequently, this adaptive process involves the recruitment of inflammatory cells [56], tissue revascularization, and extracellular matrix (ECM) reorganization to accommodate structural expansion while supporting nutrient oxidation and utilization [57, 58]. In addition, inflammation in adipose tissue, which accompanies WAT expansion, may confer beneficial effects under certain conditions [56–61]. Mammalian studies demonstrated that inflammatory responses constitute an essential process for WAT expansion, with dynamic interactions between immune cells and WAT occurring under both physiological and pathological conditions [56, 59]. In lean mice, adipose tissue-resident immune cells predominantly consist of M2 macrophages, Th2, and Treg lymphocytes, whereas obese mice exhibit a phenotypic shift toward recruitment of M1 macrophages and Th1 lymphocytes [59]. Similarly, human studies revealed that WAT from obese individuals harbors increased populations of macrophages and T lymphocytes compared to lean counterparts [56]. However, limited evidence exists in fish species. The present study indicated that inflammation-associated effects accompanying adipose tissue expansion are alleviated concurrent with reduced expansion, suggesting that immune cell populations residing in Nile tilapia adipose tissue may promote tissue expansion through inflammation-mediated mechanisms. Under physiological conditions, such immune cell-induced inflammatory responses appear to be tightly regulated, remaining within tolerable thresholds that prevent pathological obesity development in tilapia under HFD.

The above findings suggested that adipose tissue in tilapia was highly plastic in nature and that altered lipid partitioning patterns in mesenteric adipose tissue during prolonged HFD intake relieved hepatopancreatic lipid overload. The differentiation of adipocytes and the development of adipose tissue are complex processes involving the spatiotemporal-specific expression of several transcription factors and changes in metabolic profiles [21, 55, 62]. In mammals, the development of adipocytes is subdivided into five stages: pluripotent stem cell stage, preadipocyte stage, mitotic clonal expansion stage, terminal differentiation stage, and mature adipocyte stage [55]. However, research investigating alterations in pivotal transcription factors and metabolic profiles during the development of adipose tissue in fish is limited [27]. In the present study, the expression of *c/ebpγ*, a key factor for preadipocyte proliferation, was initially higher in mesenteric adipose tissue, concomitant with lower GLU metabolism and lipolysis, in fish fed HFD compared to fish fed NFD. It was reported that feeding HFD led to increased expression of *pparγ* and a number of Pparγ target genes involved in adipocyte differentiation and lipid storage [63, 64]. In the present study, the expression of *pparγ* that plays a pivotal role in the differentiation of adipocytes and adipogenesis, was elevated in mesenteric adipose tissue after feeding HFD for 8 weeks. This was accompanied by increased inflammation and lipid and GLU metabolism, which is a phase during adipose tissue undergoing significant expansion, consistent with previous research that metabolic rate increased during development of adipose tissue [63]. By 10 weeks, the expression of *pparγ* along with inflammation and lipid and GLU metabolism were all reduced in adipose tissue. In contrast, the expression of *c/ebpγ* increased and the expression of *pparγ* decreased, with the development of mesenteric adipose tissue in tilapia fed NFD. Additionally, the capacity of adipocytes to take up and metabolize GLU decreased progressively during the development of adipose tissue. Thus, the developmental pattern of tilapia adipose tissue was influenced by the lipid content of the diet, with a corresponding decrease in the metabolism of GLU in adipose tissue.

The present study demonstrated that Nile tilapia fed a HFD for 10 weeks alleviated hepatopancreatic lipid overload through mesenteric adipose tissue development and enhanced GLU metabolic preference. However, due to the relatively short experimental period, we were unable to investigate outcomes beyond this timeframe and, therefore, the mechanism of adaptation of tilapia to HFD beyond 10 weeks of feeding requires further investigation. Currently, we can only speculate on potential scenarios: it is possible that sustained adipose tissue expansion might eventually exceed adipocyte storage capacity, leading to adipocyte rupture and subsequent release of inflammatory cytokines. Such pathological changes could exacerbate the detrimental effects of HFD feeding by triggering systemic inflammation. Alternatively, ongoing HFD feeding might reinforce this adaptive mechanism, potentially accompanied by additional hepatopancreatic adaptations to lipid overload, thereby maintaining metabolic homeostasis under prolonged HFD conditions. However, the exact consequences of longer-term HFD feeding on the hepatopancreatic health of tilapia remain unclear. Therefore, the question of whether chronic HFD exposure ultimately leads to metabolic disorders or allows for sustained physiological adaptation requires more study with extended periods of feeding.

## 5. Conclusions

The present study indicated that Nile tilapia exhibited adaptations to HFD intake that involved altered lipid distribution underpinned by the development of mesenteric adipose tissue, as well as modified hepatopancreatic lipid and GLU metabolism. Feeding HFD for up to 6 weeks resulted in hepatopancreatic damage caused by lipid accumulation. This damage decreased with increasing duration of HFD feeding due to the expansion of mesenteric adipose tissue in 8–10 weeks that altered lipid distribution pattern, resulting in increased lipid accumulation in the mesenteric adipose tissue and reduced lipid in the hepatopancreas. Another potential mechanism associated with reduced NEFA pool in hepatopancreas was possibly increased transportation and metabolism of GLU, which facilitated increased lipid storage in expanded adipose tissue and mitigated lipid overload-induced hepatopancreatic vacuolization and inflammation. Adipose tissue displayed two distinct expansion strategies in tilapia fed NFD and HFD, with a switch from hypertrophy to hyperplasia possibly facilitating adipose tissue expansion in prolonged HFD feeding. The findings suggested that modulating lipid partitioning dynamics under HFD conditions and promoting GLU-centric energy utilization may constitute effective therapeutic strategies for mitigating hepatic injury under dietary lipid-overload in teleosts. Overall, the present study has provided new information for understanding the mechanism of adipose tissue development and its role in adapting to HFD to maintain metabolic homeostasis in fish.

## Figures and Tables

**Table 1:** Ingredients, formulations, and proximate compositions (% of dry weight) of the experimental diets.

Ingredients	NFD	HFD
Fishmeal^a^	8.0	8.0
Soybean meal^b^	45.0	45.0
Corn starch^c^	18.5	18.5
Distillers dried grains with solubles^d^	5.0	5.0
Bran^e^	10.0	10.0
Microcrystalline cellulose	6.0	—
Soybean oil	3.0	9.0
Monocalcium phosphate	1.5	1.5
NaCl	0.5	0.5
Vitamin and mineral premix	2.0	2.0
Choline chloride	0.5	0.5
Proximate composition (% of dry weight)		
Moisture	10.2	9.8
Crude protein	32.2	32.0
Crude lipid	5.6	12.3
Ash	7.0	6.8

*Note:* Vitamin and mineral premix (mg/kg diet): vitamin A 75 mg, vitamin D_3_ 0.065 mg, vitamin E 12,500 mg, vitamin K_3_ 500 mg, vitamin B_1_ 2500 mg, vitamin B_2_ 2500 mg, vitamin B_6_ 2500 mg, vitamin B_12_ 2.5 mg, inositol 12,500 mg, pantothenic acid 5000 mg, choline 5000 mg, niacin 12,500 mg, folic acid 500 mg, biotin 125 mg, vitamin C 5000 mg, calcium carbonate 157,000 mg, potassium dihydrogen phosphate 234,650 mg, magnesium sulfate heptahydrate 73,700 mg, sodium chloride 24,900 mg, iron gluconate 5450 mg, manganese sulfate monohydrate 1560 mg, zinc sulfate heptahydrate 2335 mg, copper sulfate pentahydrate 310 mg, potassium iodide 80 mg, cobalt chloride hexahydrate 40 mg, ammonium molybdate 30 mg, sodium selenite 10 mg.

^a^Fishmeal: crude protein 66.2%, crude lipid 9.3%.

^b^Soybean meal: crude protein 53.8%, crude lipid 2.1%.

^c^Corn starch: crude protein 0.4%, crude lipid 1.1%.

^d^Distillers dried grains with solubles: crude protein 28.4%, crude lipid 8.6%.

^e^Bran: crude protein 15.8%, crude lipid 4.9%.

**Table 2:** Growth performance and feed utilization efficiency of Nile tilapia fed normal-fat (NFD) and high-fat (HFD) diets for 6, 8, and 10 weeks (means ± SEM, *n* = 3).

Item	Lipid (%)	Time (weeks)	FBW (g)	WGR (g)	FI (%)	SGR (%/day)	FCR (%)
NFD-6 weeks	6	6	61.99 ± 2.86^cd^	209.64 ± 14.29^cd^	2.83 ± 0.22^ab^	2.69 ± 0.11^a^	1.76 ± 0.11^b^
NFD-8 weeks	6	8	75.88 ± 2.19^b^	279.02 ± 10.95^b^	2.52 ± 0.13^bc^	2.38 ± 0.05^bc^	1.91 ± 0.08^a^
NFD-10 weeks	6	10	99.05 ± 2.58^a^	394.75 ± 12.91^a^	2.09 ± 0.09^d^	2.28 ± 0.04^c^	1.83 ± 0.06^ab^
HFD-6 weeks	12	6	60.43 ± 3.53^d^	202.00 ± 17.65^d^	2.91 ± 0.17^a^	2.62 ± 0.14^ab^	1.84 ± 0.17^ab^
HFD-8 weeks	12	8	69.59 ± 2.55^bc^	247.78 ± 12.76^bc^	2.74 ± 0.17^ab^	2.22 ± 0.06^c^	2.16 ± 0.11^ab^
HFD-10 weeks	12	10	93.81 ± 1.73^a^	368.82 ± 8.67^a^	2.20 ± 0.07^cd^	2.21 ± 0.03^c^	1.96 ± 0.05^ab^
Means of main effect							
Lipid (%)							
6	—	—	78.94 ± 5.55	294.30 ± 27.77	2.48 ± 0.12	2.45 ± 0.07	1.83 ± 0.05
12	—	—	74.61 ± 5.15	272.86 ± 25.79	2.62 ± 0.12	2.35 ± 0.08	1.99 ± 0.08
Time (weeks)							
6	—	—	61.21 ± 2.06^C^	205.90 ± 10.31^C^	2.87 ± 0.10^A^	2.66 ± 0.08^A^	1.80 ± 0.09
8	—	—	72.74 ± 2.06^B^	263.52 ± 10.30^B^	2.63 ± 0.18^A^	2.30 ± 0.05^B^	2.04 ± 0.08
10	—	—	96.42 ± 1.82^A^	382.86 ± 9.09^A^	2.15 ± 0.10^B^	2.25 ± 0.03^B^	1.90 ± 0.04
Two-way ANOVA (*p*-value)							
Lipid	—	—	0.573	0.573	0.124	0.365	0.111
Time	—	—	<0.01	<0.01	<0.01	<0.05	0.054
Interaction	—	—	<0.05	<0.05	0.767	<0.05	<0.05

Abbreviations: FBW, final body weight; FCR, feed conversion ratio; FI, feed intake; SGR, specific growth rate; WGR, weight gain rate.

^a–e^Different lowercase superscript letters within a column indicate significant differences (*p* < 0.05) among the treatments by Duncan's comparison test.

^A–C^Different uppercase superscript letters within a column indicate significant differences (*p* < 0.05) among feeding durations by Duncan's comparison test.

**Table 3:** Biometric indices of Nile tilapia fed normal-fat (NFD) and high-fat (HFD) diets for 6, 8, and 10 weeks (means ± SEM, *n* = 3).

Item	Lipid (%)	Time (weeks)	VSI (%)	HSI (%)	MFI (%)	CF (g/cm^3^)
NFD-6 weeks	6	6	12.70 ± 0.31^b^	3.12 ± 0.10^b^	0.88 ± 0.03^e^	2.37 ± 0.07
NFD-8 weeks	6	8	12.67 ± 0.16^b^	2.38 ± 0.07^c^	0.87 ± 0.06^e^	2.28 ± 0.09
NFD-10 weeks	6	10	11.03 ± 0.41^c^	2.44 ± 0.13^c^	1.42 ± 0.04^c^	2.14 ± 0.04
HFD-6 weeks	12	6	12.90 ± 0.43^b^	4.09 ± 0.08^a^	1.18 ± 0.02^d^	2.31 ± 0.11
HFD-8 weeks	12	8	14.10 ± 0.27^a^	2.96 ± 0.06^b^	2.02 ± 0.06^b^	2.38 ± 0.05
HFD-10 weeks	12	10	12.17 ± 0.20^b^	3.25 ± 0.34^b^	2.34 ± 0.06^a^	2.33 ± 0.02
Means of main effect						
Lipid (%)						
6	—	—	12.14 ± 0.32	2.65 ± 0.13	1.05 ± 0.09	2.26 ± 0.05
12	—	—	13.06 ± 0.32	3.44 ± 0.20*⁣*^*∗*^	1.85 ± 0.17*⁣*^*∗*^	2.34 ± 0.04
Time (weeks)						
6	—	—	12.80 ± 0.24^A^	3.61 ± 0.22^A^	1.03 ± 0.07^B^	2.34 ± 0.06
8	—	—	13.39 ± 0.35^A^	2.67 ± 0.14^B^	1.45 ± 0.26^AB^	2.33 ± 0.05
10	—	—	11.60 ± 0.33^B^	2.85 ± 0.24^B^	1.88 ± 0.21^A^	2.24 ± 0.05
Two-way ANOVA (*p*-value)						
Lipid	—	—	0.059	<0.01	<0.001	0.227
Time	—	—	<0.05	<0.05	<0.05	0.231
Interaction	—	—	<0.05	<0.05	<0.05	0.051

Abbreviations: CF, condition factor; HSI, hepatosomatic index; MFI, mesenteric fat index; VSI, viscerosomatic index.

*⁣* ^*∗*^Asterisks indicate significant differences (*p* < 0.05) between 6% and 12% lipid levels by independent sample Student's *t*-tests.

**Table 4:** Serum biochemical parameters of Nile tilapia fed normal-fat (NFD) and high-fat (HFD) diets for 6, 8, and 10 weeks (means ± SEM, *n* = 3).

Item	Lipid (%)	Time (weeks)	TG (mmol/L)	TC (mmol/L)	NEFA (mmol/L)	GLU (mmol/L)	AST (mmol/L)	ALT (mmol/L)
NFD-6 weeks	6	6	2.45 ± 0.20^c^	2.45 ± 0.05^c^	0.10 ± 0.01^c^	5.65 ± 0.04^c^	18.97 ± 0.21^c^	16.97 ± 0.32^c^
NFD-8 weeks	6	8	3.17 ± 0.05^b^	3.12 ± 0.06^b^	0.14 ± 0.01^b^	5.98 ± 0.05^c^	12.94 ± 2.43^c^	14.71 ± 0.55^cd^
NFD-10 weeks	6	10	3.34 ± 0.23^b^	3.28 ± 0.12^b^	0.08 ± 0.01^d^	8.19 ± 0.34^a^	9.32 ± 0.25^c^	13.41 ± 0.36^d^
HFD-6 weeks	12	6	5.01 ± 0.08^a^	4.98 ± 0.04^a^	0.16 ± 0.01^a^	4.78 ± 0.04^d^	72.60 ± 9.73^a^	84.33 ± 1.45^a^
HFD-8 weeks	12	8	3.16 ± 0.49^b^	3.13 ± 0.14^b^	0.14 ± 0.01^b^	5.51 ± 0.11^c^	31.86 ± 1.03^b^	86.66 ± 1.54^a^
HFD-10 weeks	12	10	2.64 ± 0.05^c^	2.63 ± 0.12^c^	0.09 ± 0.01^d^	7.45 ± 0.23^b^	17.68 ± 1.64^c^	53.52 ± 1.32^b^
Means of main effect								
Lipid (%)								
6	—	—	2.99 ± 0.16	2.95 ± 0.13	0.11 ± 0.01	6.61 ± 0.41	13.74 ± 1.57	15.02 ± 0.56
12	—	—	3.60 ± 0.36	3.58 ± 0.36	0.13 ± 0.01	5.91 ± 0.40	40.72 ± 8.71*⁣*^*∗*^	74.83 ± 5.39*⁣*^*∗*^
Time (weeks)								
6	—	—	3.73 ± 0.58	3.72 ± 0.57	0.13 ± 0.01^A^	5.22 ± 0.20^B^	45.78 ± 12.76^A^	50.65 ± 15.08
8	—	—	3.16 ± 0.03	3.12 ± 0.07	0.14 ± 0.01^A^	5.74 ± 0.12^B^	22.40 ± 4.39^AB^	50.69 ± 16.11
10	—	—	2.99 ± 0.19	2.95 ± 0.16	0.08 ± 0.01^B^	7.82 ± 0.25^A^	13.50 ± 2.01^B^	33.46 ± 8.99
Two-way ANOVA (*p*-value)								
Lipid	—	—	0.139	0.112	0.154	0.246	<0.01	<0.001
Time	—	—	0.181	0.156	<0.01	<0.01	<0.05	0.415
Interaction	—	—	<0.05	<0.05	<0.05	<0.05	<0.05	<0.05

Abbreviations: ALT, alanine aminotransferase; AST, aspartate aminotransferase; GLU, glucose; NEFA, nonesterified fatty acid; TC, total cholesterol; TG, triglyceride.

**Table 5:** Contents of biochemical parameters in hepatopancreas of Nile tilapia fed normal-fat (NFD) and high-fat (HFD) diets for 6, 8, and 10 weeks (means ± SEM, *n* = 3).

Item	Lipid (%)	Time (weeks)	TG (mmol/gprot)	TC (mmol/gprot)	NEFA (mmol/gprot)	GLU (mmol/gprot)	MDA (mmol/gprot)
NFD-6 weeks	6	6	0.33 ± 0.01^c^	0.09 ± 0.01^c^	0.21 ± 0.01^b^	1.55 ± 0.07^b^	0.34 ± 0.02^b^
NFD-8 weeks	6	8	0.31 ± 0.01^c^	0.09 ± 0.01^c^	0.15 ± 0.01^bc^	1.62 ± 0.16^b^	0.36 ± 0.01^b^
NFD-10 weeks	6	10	0.30 ± 0.03^c^	0.08 ± 0.01^c^	0.18 ± 0.01^bc^	1.68 ± 0.01^b^	0.38 ± 0.01^b^
HFD-6 weeks	12	6	0.69 ± 0.01^a^	0.13 ± 0.01^a^	0.38 ± 0.04^a^	1.84 ± 0.04^a^	0.73 ± 0.22^a^
HFD-8 weeks	12	8	0.57 ± 0.01^b^	0.08 ± 0.01^c^	0.15 ± 0.01^c^	1.67 ± 0.14^b^	0.31 ± 0.02^b^
HFD-10 weeks	12	10	0.65 ± 0.02^a^	0.11 ± 0.01^b^	0.35 ± 0.04^a^	2.04 ± 0.08^a^	0.42 ± 0.05^b^
Means of main effect							
Lipid (%)							
6	—	—	0.32 ± 0.01	0.09 ± 0.01	0.18 ± 0.01	1.62 ± 0.05	0.36 ± 0.01
12	—	—	0.64 ± 0.02*⁣*^*∗*^	0.11 ± 0.01*⁣*^*∗*^	0.30 ± 0.05*⁣*^*∗*^	1.85 ± 0.07*⁣*^*∗*^	0.49 ± 0.09
Time (weeks)							
6	—	—	0.51 ± 0.10	0.11 ± 0.01^A^	0.30 ± 0.05^A^	1.69 ± 0.07	0.54 ± 0.13
8	—	—	0.44 ± 0.07	0.09 ± 0.01^B^	0.15 ± 0.01^B^	1.64 ± 0.10	0.33 ± 0.02
10	—	—	0.48 ± 0.10	0.10 ± 0.01^AB^	0.26 ± 0.05^A^	1.86 ± 0.09	0.40 ± 0.02
Two-way ANOVA (*p*-value)							
Lipid		—	<0.001	<0.05	<0.05	<0.05	0.182
Time		—	0.591	<0.05	<0.05	0.116	0.096
Interaction		—	<0.05	<0.05	<0.05	<0.05	<0.05

Abbreviations: GLU, glucose; MDA, malondialdehyde; NEFA, nonesterified fatty acid; TC, total cholesterol; TG, triglyceride.

## Data Availability

The data that support the findings of this study are available from the corresponding author upon reasonable request.

## Supporting Information

Supporting Information This section collects primer sequences and muscle tissue analyses, including measurements of crude fat content and gene expression levels.
