# Supplementary material for: Adipocyte Hyperplasia Facilitated Adipose Tissue Expansion to Alleviate Hepatopancreas Injury in Nile Tilapia (Oreochromis niloticus) Fed High-Fat Diet
Source: Aquac Nutr. 2025 Jul 2;2025:1260555. doi: 10.1155/anu/1260555 (PMC12240657; doi:10.1155/anu/1260555)
Supplement: Supporting Information — This section collects primer sequences and muscle tissue analyses, including measurements of crude fat content and gene expression levels. [file 1260555.f1.docx]

# Adipocyte hyperplasia facilitated adipose tissue expansion to alleviate hepatopancreas injury in Nile tilapia (*Oreochromis niloticus*) fed high-fat diet

Senyue Tan^a^, Jiamin Wei^a^, Ailan Diao^a^, Douglas R. Tocher^a, b^, Zeling Lin^a^, Bing Chen^c^, Ruixin Li^a^, Shuqi Wang^a*^, Cuiying Chen^a*^

^a^ Guangdong Provincial Key Laboratory of Marine Biotechnology, Institute of Marine Sciences, Shantou University, Shantou 515063, China

^b^ Institute of Aquaculture, Faculty of Natural Sciences, University of Stirling, Stirling FK9 4LA, Scotland, UK

^c^ Institute of Animal Science, Guangdong Academy of Agricultural Sciences, Collaborative Innovation Center of Aquatic Sciences, Key Laboratory of Animal Nutrition and Feed Science in South China, Guangzhou 510640, China.

*Corresponding Author

Cuiying Chen, Ph.D. (E–mail: cychen@stu.edu.cn)

Shuqi Wang, Ph.D. (E–mail: sqw@stu.edu.cn)

Present address of the corresponding author: Institute of Marine Sciences, Shantou University, 243 DaXue Road, Shantou 515063, China.

**Supplementary Table**

Table S1. Primers used for qPCR analysis.

|  | Gene name | Sense and antisense primer (5′–3′) | GenBank no. |
| --- | --- | --- | --- |
| Internal reference | *18s rRNA* | F: TTCAGCCACACGAGATTGAG  R: CCGAGGACCTCACTAAACCA | XR_003219985.1 |
| Pro-inflammatory | *tnfα* | F: GTTGTTAACAGGCGTGCTGA  R: TGAGCTCTAGTCCTCCCTGA | XM_025902124.1 |
| Pro-inflammatory | *tnfβ* | F: GCCTCACAATTCTCAGCCAC  R: AAACACGCCAAAGAAGGTCC | NM_001279533.1 |
| Pro-inflammatory | *il-1β* | F: AATGAAGCGTGTGGTCAACC  R: GCTCCTCTTTTGGCTGTCAG | XM_019365842.2 |
| Lipogenesis | *accα* | F: TAGCTGAAGAGGAGGGTGCAAGA  R: AACCTCTGGATTGGCTTGAACA | XM_005471970 |
| Lipogenesis | *fas* | F: ACATCGGCAAGGTCCTGGTTCA  R: GGCAGAAGGTGCGACAGATAGC | XM_003454056 |
| Lipogenesis | *dgat1* | F: ATGACTGAGAGACTCCTGCG  R: TCCTCAGAAGCGGCTTGTAA | XM_003458972 |
| Lipogenesis | *srebp1* | F: TCCACCACAGACGCAGACACA  R: AATGGTCGGCGGTGAGGATGA | XM_005471970 |
| Lipids transport | *fatp4* | F: GTGAGGAAGAGCAGGGACAA  R: TGCTATCCTCATCTGCAGCT | XM_005451375 |
| Lipids transport | *fabp1* | F: TGGTCGCAGTGAGGACATAC  R: AACACAGGCACGGAAGAGT | XM_003446092 |
| Lipid mobilization | *pparα* | F: GCACGGCTCATACTCACTGGAA  R: TCCGAGGACGCTGTCAGACT | NM_001290066 |
| Lipolysis | *hsl* | F: GCCCGTTACATTGCCTCCAGTT  R: GCCGCAGTCGTTGTCGTTGA | XM_005463937.4 |
| Lipolysis | *lpla* | F: ATTGCCGGAGACCTTACCAA  R: TGGTCTCTGGATGCCGATAC | NM_001279753 |
| Lipolysis | *cpt1b* | F: TTCACTGCCTCACTGACTCAA  R: TGGAAGCCTACTGCCTGATG | XM_003440552 |
| Lipolysis | *atgl* | F: AAAACGTCCTGGTGACCCCAGT  R: TAGGAGGAATGATGCCACAGTACA | XM_003440346.5 |
| Lipolysis | *adipor2* | F: ATTCCTCACGACGTACTCCC  R: AACATCCCGATCACCACCTT | XM_025899627 |
| Glycolysis | *pfk* | F: TTGCCAACTCACCAGAAACG  R: GAGCGTCGAGTCACATGTTC | XM_003447353 |
| Glycolysis | *hk* | F: GGGATAACAAAGCTGCCGAG  R: TTTGGTTGGCAAGTCGGAAG | XM_003454508 |
| Gluconeogenesis | *g6pase* | F: CAGCAGTTCCCCATCACATG  R: AGACCCTGGACATGCATACC | XM_003448671.4 |
| Gluconeogenesis | *pepck* | F: TGCTCATCCCAACTCTCGTT  R: ACCATGCTGCCAGTCAAAAG | XM_003448375 |
| Pentose phosphate metabolism | *g6pd* | F: CATCCCTCTCTCTCGCTCTG  R: CGGGTAGTAGGCCATCTCTG | XM_013275691 |
| Glucose transport | *glut2* | F: CATTGGCATTCTAATCAGCCAGGT  R: TTGTAATATTGCTGGCGCTCCA | XM_003442884.5 |
| Preadipocyte differentiation | *c/ebpγ* | F: GTGAGGAAGAGCAGGGACAA  R: TGCTATCCTCATCTGCAGCT | XM_005465067 |
| Preadipocyte differentiation | *pparγ* | F: GCAGCACCAGACCTCCACTCTT  R: TGTCCACCGAAGCCCGTCATT | NM_001290200 |

*18S rRNA*, 18S ribosomal RNA; *accα*, acetyl-CoA carboxylase alpha; *atgl*, adipose triglyceride lipase; *adipor2*, adiponectin receptor 2; *pfk*, phosphofructokinase; *c/ebpγ*, CCAAT/enhancer binding protein gamma; *cpt1b*, carnitine palmitoyl transferase 1b; *dgat1*, diacylglycerol O-acyltransferase 1; *fabp1*, fatty acid binding protein 1; *fas*, fatty acid synthase;  *fatp4*, fatty acid transport protein 4; *g6pase*, glucose-6-phosphatase; *g6pd*, glucose-6-phosphate dehydrogenase; *glut2*, glucose transport 2; *hk*, hexokinase; *hsl*, hormone-sensitive triglyceride lipase; *il-1β*, interleukin-1 beta; *lpla*, lipoprotein lipase alpha; *srebp1*, sterol regulatory element-binding protein 1; *tnfα*, tumor necrosis factor alpha; *tnfβ*, tumor necrosis factor beta; *pepck*, phosphoenolpyruvate carboxy kinase; *pparα*, peroxisome proliferator-activated receptor alpha; *pparγ*, peroxisome proliferator-activated receptor gamma.

**Supplementary Figures**

Figure S1


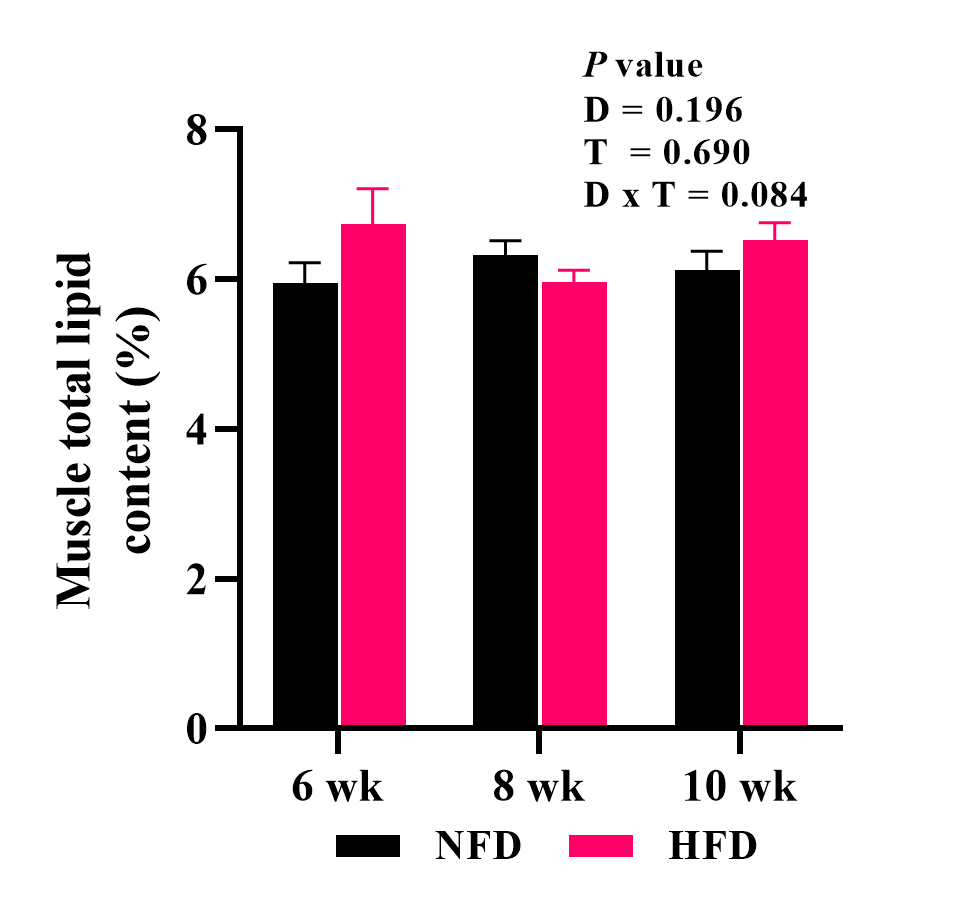


Total lipid contents of dorsal muscle of Nile tilapia fed normal (NFD) and high (HFD) diets for 6, 8 and 10 weeks. Data are presented as means ± SEM (*n* = 3).

Figure S2


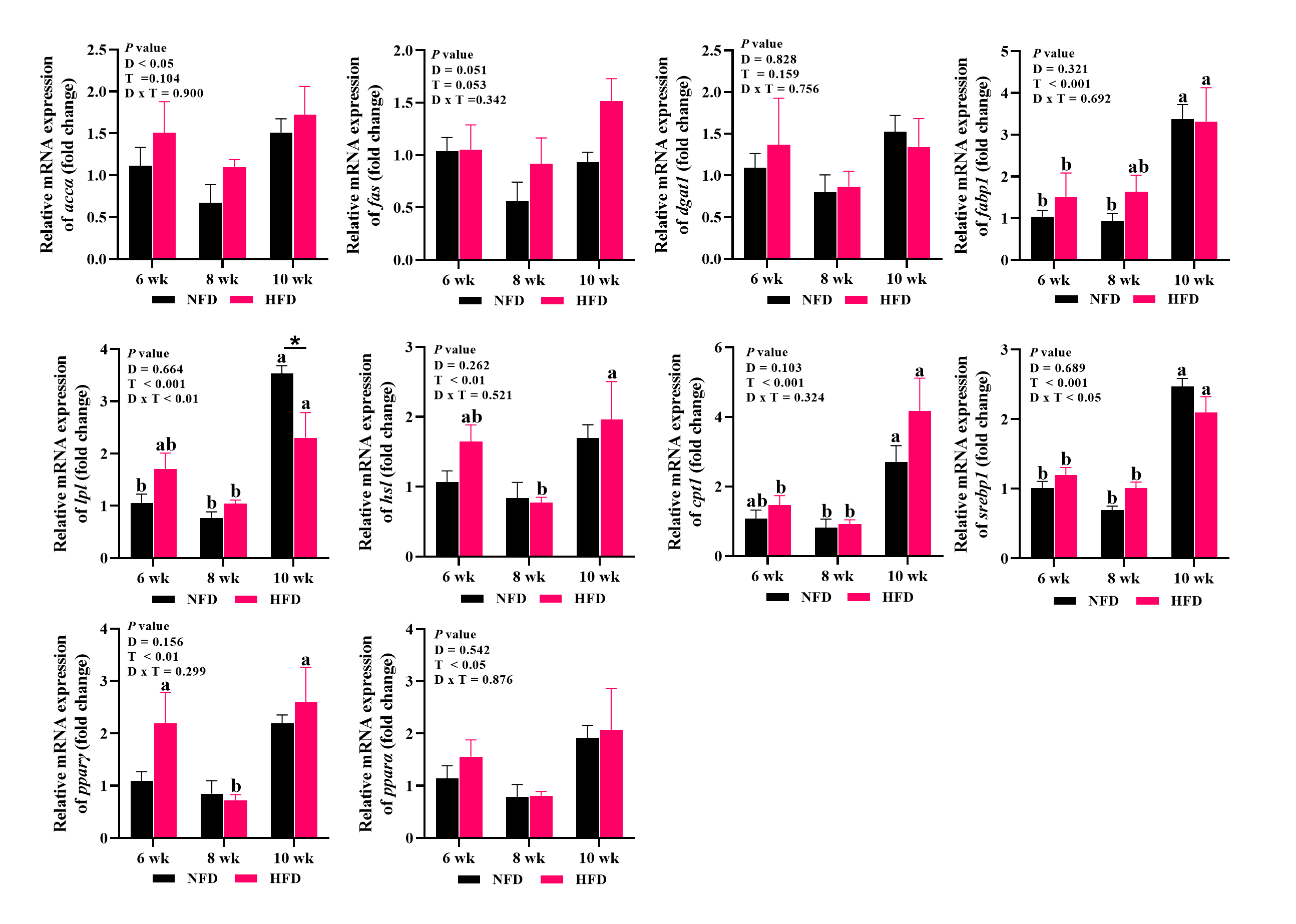


Relative expression levels of genes related to lipid metabolism in dorsal muscle of Nile tilapia fed NFD and HFD for 6-, 8- and 10-wk. Data are presented as means ± SEM (*n* = 3). Asterisks indicate a significant difference between fish fed HFD and fish fed NFD, ^∗^*P* < 0.05, ^∗∗^*P* < 0.01. Different letters on columns for the same diet indicate significant differences due to duration of feeding (*P* < 0.05). *accα*: acetyl-CoA carboxylase alpha; *cpt1*: carnitine palmitoyl transferase 1; *dgat1*: diacylglycerol O-acyltransferase 1; *fas*: fatty acid synthase; *fabp1*: fatty acid binding protein 1; *hsl*: hormone-sensitive triglyceride lipase; *lpl*: lipoprotein lipase; *pparα*: peroxisome proliferator-activated receptor alpha; *pparγ*: peroxisome proliferator-activated receptor gamma; *srebp1*: sterol regulatory element-binding protein 1.
